# Efficacy and safety of low-dose thrombolysis in intermediate-risk pulmonary embolism: a meta-analysis

**DOI:** 10.3389/fcvm.2026.1862385

**Published:** 2026-08-13

**Authors:** Chen Zhu, Wei Chen, Lingcong Wang, Xi Xing

**Affiliations:** Intensive Care Unit, The First Affiliated Hospital of Zhejiang Chinese Medical University (Zhejiang Provincial Hospital of Chinese Medicine), Hangzhou, China

**Keywords:** intermediate-risk pulmonary embolism, low-dose thrombolysis, meta-analysis, mortality, safety

## Abstract

**Objective:**

To systematically evaluate the efficacy and safety of low-dose thrombolysis in patients with intermediate-risk pulmonary embolism (PE).

**Methods:**

A systematic search was conducted in PubMed, Embase, and the Cochrane Library up to December 6, 2025. Randomized controlled trials (RCTs), non-randomized controlled studies, and cohort studies investigating low-dose thrombolysis for intermediate-risk PE were included. Two researchers independently screened the literature, extracted data, and assessed study quality. A meta-analysis was performed using Stata software (version 16.0) to calculate pooled estimates of mortality, bleeding incidence, and recurrence rates. The efficacy and safety of low-dose thrombolysis were compared with those of anticoagulation alone or full-dose thrombolysis.

**Results:**

This meta-analysis included 13 studies (1,018 patients with intermediate-risk PE). Low-dose thrombolysis yielded a 1-month and 1-year mortality of 2% (95% CI: 0.00–0.03; 0.00–0.05), recurrence of 1% (95% CI: 0.00–0.02), major bleeding of 1% (95% CI: 0.00–0.03), and minor bleeding of 7% (95% CI: 0.05–0.10). Subgroup analysis showed ultrasound-assisted catheter-directed thrombolysis (USAT) had 1-month and 1-year mortality rates of 2% (95% CI: 0.01–0.04) and 1% (95% CI: 0.00–0.03), respectively, vs. 1% (95% CI: 0.00–0.03) and 7% (95% CI: 0.01–0.16) for low-dose systemic thrombolysis. Compared to anticoagulation alone or full-dose thrombolysis, low-dose systemic thrombolysis showed no significant differences in mortality or recurrence, but had lower bleeding risk than full-dose thrombolysis (RD = −0.23, 95% CI: −0.44 to −0.01). USAT significantly reduced 1-month mortality vs. anticoagulation alone (RD = −0.09, 95% CI: −0.13 to −0.04); however, this stems from a single non-randomized study requiring RCT validation. Bleeding rates showed no significant difference.

**Conclusion:**

In patients with intermediate-risk PE, low-dose thrombolysis yields low mortality and recurrence rates with an acceptable bleeding risk. Compared with full-dose thrombolysis, it offers a safety advantage by reducing the incidence of bleeding; however, its comparative efficacy regarding mortality and recurrence remains undetermined and may be equivalent. Further high-quality RCTs are warranted.

**Systematic Review Registration:**

https://www.crd.york.ac.uk/PROSPERO/view/CRD420261284475.

## 1 Introduction

Acute pulmonary embolism (PE) is a leading cause of pulmonary circulatory failure and cardiovascular mortality. Based on hemodynamic status and markers of myocardial strain, PE is classified into three categories: high-risk (hemodynamically unstable), intermediate-risk (hemodynamically stable with evidence of right ventricular [RV] dysfunction), and low-risk (hemodynamically stable without RV dysfunction) (1). Patients with intermediate-risk PE represent a primary focus of clinical management; while their short-term mortality rate is lower than that of high-risk patients, it remains significantly higher than that of the low-risk group. Furthermore, these patients are at a substantial risk of progressive RV dysfunction and rapid hemodynamic deterioration (2, 3). Although conventional anticoagulation prevents thrombus extension, it has limited efficacy in achieving rapid thrombus resolution or preventing clinical worsening (1, 3). Consequently, identifying a therapeutic strategy that effectively dissolves thrombi and improves hemodynamics while minimizing the risk of major bleeding remains a critical challenge in the management of PE.

Full-dose systemic thrombolysis (e.g., 100 mg of rt-PA administered over 2 h) has been shown to significantly reduce mortality in high-risk patients with PE. However, its routine use in the intermediate-risk population is constrained by the heightened risk of major hemorrhage and intracranial bleeding (4, 5). Recently, low-dose thrombolytic regimens (e.g., 30–50 mg of rt-PA) have been proposed as a safer alternative to balance efficacy and safety. Preliminary studies suggest that low-dose regimens can improve RV function and reduce pulmonary arterial pressure without substantially increasing the risk of major bleeding complications (5, 6). Nevertheless, evidence regarding its impact on mortality, its comparative advantages over anticoagulation alone, and the relative safety across different low-dose delivery strategies remains inconsistent (1, 3). While some meta-analyses suggest potential benefits, they often conclude that the evidence is insufficient; others indicate a possible reduction in short-term mortality with an acceptable safety profile (4, 5).

To address these unresolved clinical questions, this study provides a systematic review and meta-analysis of the current evidence. We evaluated the clinical efficacy (including mortality and recurrence rates) and safety (primarily bleeding events) of low-dose thrombolysis in patients with intermediate-risk PE. This work aims to synthesize the currently available evidence concerning low-dose thrombolysis strategies and outcome assessments,providing a preliminary reference to inform the design of future rigorous clinical trials and to highlight areas requiring further investigation, rather than serving as a definitive basis for current guideline changes.

## 2 Materials and methods

### 2.1 Registration of the meta-analysis

This systematic review and meta-analysis were conducted in accordance with the Preferred Reporting Items for Systematic Reviews and Meta-Analyses (PRISMA) statement. The study protocol was registered in the International Prospective Register of Systematic Reviews (PROSPERO) to ensure transparency and minimize the risk of selective reporting bias. The registration number is CRD420261284475 (Date created: January 13 2026).

### 2.2 Search strategy

Two investigators independently developed and executed the search strategy across PubMed, Embase, and the Cochrane Library from their inception through December 6, 2025. The search employed a combination of Medical Subject Headings (MeSH) and Emtree terms and free-text keywords, linked with Boolean operators (AND, OR). Core search concepts included “pulmonary embolism”, “low-dose”, and “thrombolysis/thrombolytic agents”. To ensure a comprehensive retrieval of all relevant studies, specific terms for various thrombolytic drugs—such as alteplase, urokinase, tenecteplase, streptokinase, and reteplase—were included. The detailed search strategy for PubMed is presented in Table 1, which was further adapted for Embase and the Cochrane Library according to their respective subject heading systems and field tags.

**Table 1:** Search strategy for the pubMed database.

No.	Search strategy
#1	“Pulmonary Embolism”[Mesh] OR “pulmonary embolism”[Title/Abstract] OR “lung embolism”[Title/Abstract] OR “pulmonary thromboembolism”[Title/Abstract] OR “pulmonary arterial embolism”[Title/Abstract]
#2	“low-dose”[Title/Abstract] OR “reduced-dose”[Title/Abstract] OR “half-dose”[Title/Abstract] OR “dose reduction”[Title/Abstract] OR “attenuated dose”[Title/Abstract] OR “submaximal dose”[Title/Abstract] OR “minimal dose”[Title/Abstract] OR “lower dose”[Title/Abstract]
#3	“thrombolysis”[Mesh] OR “thrombolytic therapy”[Title/Abstract] OR “fibrinolysis”[Title/Abstract] OR “fibrinolytic therapy”[Title/Abstract] OR “systemic thrombolysis”[Title/Abstract] OR “catheter-directed thrombolysis”[Title/Abstract] OR “ultrasound-assisted thrombolysis”[Title/Abstract] OR “directed thrombolysis”[Title/Abstract]
#4	“alteplase”[Mesh] OR “alteplase”[Title/Abstract] OR “rt-PA”[Title/Abstract] OR “tPA”[Title/Abstract] OR “urokinase”[Mesh] OR “urokinase”[Title/Abstract] OR “tenecteplase”[Mesh] OR “tenecteplase”[Title/Abstract] OR “streptokinase”[Mesh] OR “streptokinase”[Title/Abstract] OR “reteplase”[Mesh] OR “reteplase”[Title/Abstract] OR “nattokinase”[Title/Abstract] OR “staphylokinase”[Title/Abstract] OR “desmoteplase”[Title/Abstract] OR “fibrynase”[Title/Abstract]
#5	#3 OR #4
#6	#1 AND #2 AND #5

### 2.3 Inclusion and exclusion criteria

#### 2.3.1 Inclusion criteria

The inclusion criteria for this study were as follows: (1) Study design:Randomized controlled trials (RCTs), non-randomized controlled studies, or cohort studies (prospective or retrospective) investigating the efficacy and safety of low-dose thrombolysis for intermediate-risk pulmonary embolism. (2) Study population:Adult patients (≥18 years) diagnosed with intermediate-risk pulmonary embolism, defined according to international guidelines or established research standards as hemodynamically stable patients (without persistent hypotension or shock) presenting with right ventricular dysfunction (e.g., right ventricular dilatation or an increased right ventricle/left ventricle ratio on echocardiography or computed tomography) and/or elevated myocardial injury biomarkers (e.g., cardiac troponin, B-type natriuretic peptide [BNP], or N-terminal pro-BNP [NT-proBNP]) (7–9). Studies using the term “submassive pulmonary embolism” were considered eligible if the enrolled population corresponded to contemporary definitions of intermediate-risk PE, namely hemodynamic stability with evidence of right ventricular dysfunction and/or myocardial injury. (3) Interventions:The experimental group received low-dose thrombolysis in addition to conventional anticoagulation therapy. Thrombolysis could be administered systemically or via a catheter-directed approach using thrombolytic agents, including but not limited to alteplase, urokinase, tenecteplase, streptokinase, and reteplase, at doses lower than the guideline-recommended standard dose. For rt-PA (alteplase): Systemic low-dose was defined as a fixed total dose <100 mg (the included studies used doses ranging from 25 mg to 50 mg; the minimum systemic dose included was 25 mg). For catheter-directed administration, low-dose was defined as a total dose ≤24 mg per patient (ranging from 4 mg to 24 mg; the minimum catheter-directed dose included was 4 mg per lung). For urokinase: Systemic low-dose was defined as a fixed dose of 500,000 IU/day administered for 5–7 days. Catheter-directed low-dose was defined as an initial 200,000 IU bolus followed by a 100,000 IU/hour local infusion. Regarding weight-adjusted regimens, studies utilizing a weight-based low-dose strategy (e.g., 0.6 mg/kg of rt-PA) were considered; however, upon review of the literature, none of the 13 studies ultimately included in this meta-analysis employed a weight-adjusted “half-dose” protocol. All included studies used fixed, low absolute doses as defined above. The control group received conventional treatment alone, primarily standard anticoagulation therapy (e.g., unfractionated heparin, low-molecular-weight heparin, or direct oral anticoagulants), along with necessary supportive care. Low-dose thrombolysis was defined *a priori* as a thrombolytic regimen using a total dose lower than the conventional standard systemic dose recommended in guidelines or commonly used in clinical practice. For systemic thrombolysis, this generally refers to rt-PA doses below 100 mg. For catheter-directed thrombolysis, studies were eligible if the total thrombolytic dose was explicitly described by the authors as low-dose or substantially lower than standard systemic dosing. (4) Outcome measures:Eligible studies were required to report at least one of the following outcomes: ① Pulmonary embolism–related mortality (1-month mortality and/or 1-year mortality); ② Incidence of bleeding events (including major bleeding and/or intracranial hemorrhage). Bleeding events were classified as major or minor bleeding. Major bleeding was defined according to the criteria adopted by each original study (e.g., International Society on Thrombosis and Hemostasis [ISTH] criteria, Global Utilization of Streptokinase and Tissue Plasminogen Activator for Occluded Coronary Arteries [GUSTO] criteria, or study-specific definitions). Table 2 presents the major bleeding definition criteria from the major literature. Minor bleeding was defined as any bleeding event that did not meet the criteria for major bleeding. When a study did not distinguish between major and minor bleeding, the total reported bleeding events were extracted and analyzed as “any bleeding.” ③ Recurrent pulmonary embolism. (5) Data availability:Studies had to provide sufficient data for pooled analysis, including the number of events and total sample size per group, or sufficient information to calculate effect sizes. Studies meeting all the above criteria were included in the analysis.

**Table 2:** Overview of major bleeding definition criteria in the literature.

Study	Year	Definition of Major Bleeding
Victor F. Tapson (10)	2018	ISTH criteria: 1) Fatal bleeding; 2) Symptomatic bleeding in a critical area or organ (intracranial, intraspinal, intraocular, retroperitoneal, intra-articular, pericardial, or intramuscular with compartment syndrome); 3) Bleeding causing a decrease in hemoglobin of ≥2 g/dL or leading to transfusion of ≥2 U of whole blood or red blood cells.
Ling-Yun Zhang (5)	2018	Not explicitly defined: The text does not specify a standard definition, but categorizes safety outcomes simply as “major bleeding” and “minor bleeding” (e.g., intracranial hemorrhage was considered major).
Chuan Jiang (11)	2021	ISTH criteria: 1) Fatal bleeding; 2) Symptomatic bleeding in a critical area or organ; 3) Bleeding causing a fall of haemoglobin of 2 g/dL or more or leading to a transfusion of two or more units of packed red blood cells. Bleeding not meeting these criteria was defined as minor.
Cyrus Moini (12)	2025	ISTH criteria: The manuscript explicitly states that all hemorrhagic events were collected and classified using the International Society on Thrombosis and Haemostasis (ISTH) definition.
Xiaogang Jing (13)	2018	Not explicitly defined: The study recorded “hemorrhage” as a general adverse event but did not provide a specific, quantified definition for major bleeding.
Sara Alcántara Carmona (14)	2018	Custom criteria: Major bleeding was defined as bleeding leading to the transfusion of one to three units of packed red blood cells (PRBCs). Life-threatening bleeding was separately defined as associated with hemodynamic instability, transfusion of ≥4 PRBCs, or intracranial bleeding.
Emily Zientek (15)	2023	ISTH criteria: Defined as major bleeding indicators including brain or abdominal imaging, a decrease in hemoglobin by more than 3 g/dL over 24 h, or the requirement for blood transfusion.
Daniel P. Rothschild (16)	2019	ISTH & GUSTO criteria: Assessed using both. ISTH major bleeding as defined above. GUSTO criteria categorize “severe/life-threatening bleeding” (causing hemodynamic compromise requiring intervention or ICH) and “moderate bleeding” (requiring transfusion without hemodynamic compromise).
Emine Serap Yilmaz (17)	2021	ISTH criteria: The study explicitly states that major extracranial bleeding within 7 days was defined according to the International Society on Thrombosis and Haemostasis (ISTH) definition.
Brandon Hooks (18)	2020	GUSTO criteria: Bleeding events were assessed using the GUSTO (Global Utilization of Streptokinase and Tissue Plasminogen Activator for Occluded Coronary Arteries) criteria, categorizing into major and minor bleeding.
Sandeep Bagla (19)	2015	SIR criteria: Defined per the Society of Interventional Radiology (SIR) Reporting Standards. Minor bleeding is that requiring conservative therapy only; major bleeding requires blood transfusion, surgical intervention, or causes hemodynamic instability.
Abdelrahman Elhakim (20)	2022	GUSTO criteria: Severe or life-threatening bleeding was defined as either intracranial hemorrhage or bleeding that caused hemodynamic compromise and required intervention. Moderate bleeding required a blood transfusion without resulting in hemodynamic compromise.
Hani Al-Terki (21)	2023	GUSTO criteria: The manuscript explicitly states that the GUSTO bleeding score was used to define and evaluate major and minor bleeding events.

#### 2.3.2 Exclusion criteria

Studies were excluded if they met any of the following criteria: (1) Publication Type: Reviews, systematic reviews, meta-analyses, case reports, conference abstracts, commentaries, editorials, and studies for which the full text was unavailable. (2) Study Population: Research primarily focusing on patients with high-risk (massive) pulmonary embolism or studies involving pediatric populations (minors <18 years). (3) Interventions and Comparisons: Studies that did not clearly employ a low-dose thrombolysis regimen, those where the thrombolytic dose could not be verified as lower than the standard dose, or those with an undefined intervention in the control group. (4) Data and Reporting: Duplicate publications (in which case, only the most comprehensive version or the one with the largest sample size was retained), studies with incomplete reporting of key outcomes that precluded pooled analysis, and studies containing uncorrectable statistical errors or discrepancies.

### 2.4 Literature screening process

Two researchers (CZ and WC) independently conducted the literature screening process. Initially, bibliographic management software (e.g., NoteExpress) was used to organize the retrieved records and systematically remove duplicates. A preliminary screening was then performed by evaluating titles and abstracts to exclude studies that clearly did not meet the eligibility criteria. For all potentially relevant citations, full-text articles were retrieved and rigorously assessed to confirm inclusion in the final meta-analysis. Any disagreements encountered during the selection process were resolved through discussion and consensus between the two primary researchers; in cases of persistent disagreement, a third researcher served as arbiter. Furthermore, the reference lists of the included studies were manually searched to identify any additional relevant publications that may have been missed during the electronic database search.

### 2.5 Data extraction

Two researchers (CZ and LW) independently extracted data from the included studies using a standardized extraction form and cross-verified the results to ensure accuracy. The extracted parameters primarily included: (1) baseline study information, such as the primary author, publication year, study location, and study design; (2) participant characteristics, including the sample size per group and specific diagnostic criteria; (3) intervention details, covering the treatment regimens for both experimental and control groups, specifically the delivery method (systemic or catheter-directed), thrombolytic agent, and dosage, as well as the anticoagulation or supportive care protocols used in the control group; and (4) clinical outcomes, specifically the number of events related to PE-associated mortality, bleeding (major hemorrhage and intracranial hemorrhage), and PE recurrence. In cases where reported data were incomplete, missing values were calculated or derived from available information whenever possible. For studies with multiple publications, only the most comprehensive report or the one with the largest patient cohort was utilized.

### 2.6 Literature quality assessment

Two researchers (CZ and XX) independently appraised the methodological quality of the included studies using tools specific to each study design. For randomized controlled trials, the Cochrane Risk of Bias 2.0 (RoB 2) tool was employed to evaluate five domains: bias arising from the randomization process, deviations from intended interventions, missing outcome data, measurement of the outcome, and selection of the reported results. Each domain was categorized as “low risk of bias,” “some concerns,” or “high risk of bias.” For non-randomized controlled trials, the Risk Of Bias In Non-randomized Studies of Interventions (ROBINS-I) tool was used to assess bias across seven domains: confounding, participant selection, classification of interventions, deviations from intended interventions, missing outcome data, measurement of outcomes, and selection of the reported results. The overall risk of bias was graded as “low,” “moderate,” “serious,” or “critical.” For cohort studies, methodological quality was evaluated using the Newcastle-Ottawa Scale (NOS), which scores studies across three dimensions: selection of study groups (up to 4 points), comparability of groups (up to 2 points), and outcome assessment (up to 3 points). Any discrepancies during the appraisal process were resolved through discussion and consensus, with arbitration by a third researcher if necessary. The results of these quality assessments were subsequently utilized in sensitivity and subgroup analyses to evaluate the impact of study quality and design on the robustness of the pooled estimates.

### 2.7 Statistical methods

Statistical analysis was performed using Stata software (version MP 16.0). For dichotomous outcomes, the risk ratio (RR) or risk difference (RD), along with their 95% confidence intervals (CIs), were used as effect measures. Before pooling effect sizes, heterogeneity among studies was assessed using Cochran's *Q*-test (significance level *α* = 0.10) and the I^2^ statistic. If I^2^ ≤ 50% and *P* ≥ 0.10, heterogeneity was considered not significant, and a fixed-effects model was used for meta-analysis. If I^2^ > 50% or *P* < 0.10, heterogeneity was considered significant, and a random-effects model was employed, with exploration of potential clinical and methodological sources of heterogeneity. Subgroup analysis: To assess heterogeneity and compare different thrombolysis strategies, prespecified subgroups were defined by intervention regimen or dose.Sensitivity analysis: The robustness of the pooled results was evaluated using a leave-one-out approach, sequentially excluding each study. Publication bias assessment: For outcomes with ≥10 included studies, funnel plots were used for the qualitative evaluation. Funnel plots and formal tests for funnel-plot asymmetry were considered only when at least 10 studies were available for an outcome. Publication bias was not formally assessed for outcomes with fewer than 10 studies because of the limited power of such tests. All statistical tests were two-sided, with a *P* value < 0.05 considered statistically significant (except for the heterogeneity *Q*-test).

For dichotomous outcomes, the RR or RD, along with their 95% CIs, were used as effect measures. For comparative analyses between different treatment strategies, RD was selected as the primary effect measure. This decision was made because several subgroup comparisons (e.g., mortality comparisons involving full-dose thrombolysis) contained zero events in both arms. In such cases, RR is mathematically undefined and cannot be computed without arbitrary continuity corrections, whereas RD can be validly calculated. RD was therefore used to ensure statistical consistency across all analyses and avoid introducing correction-related bias. RR was also calculated and reported where mathematically permissible for transparency.

## 3 Results

### 3.1 Literature screening

**Figure 1:**
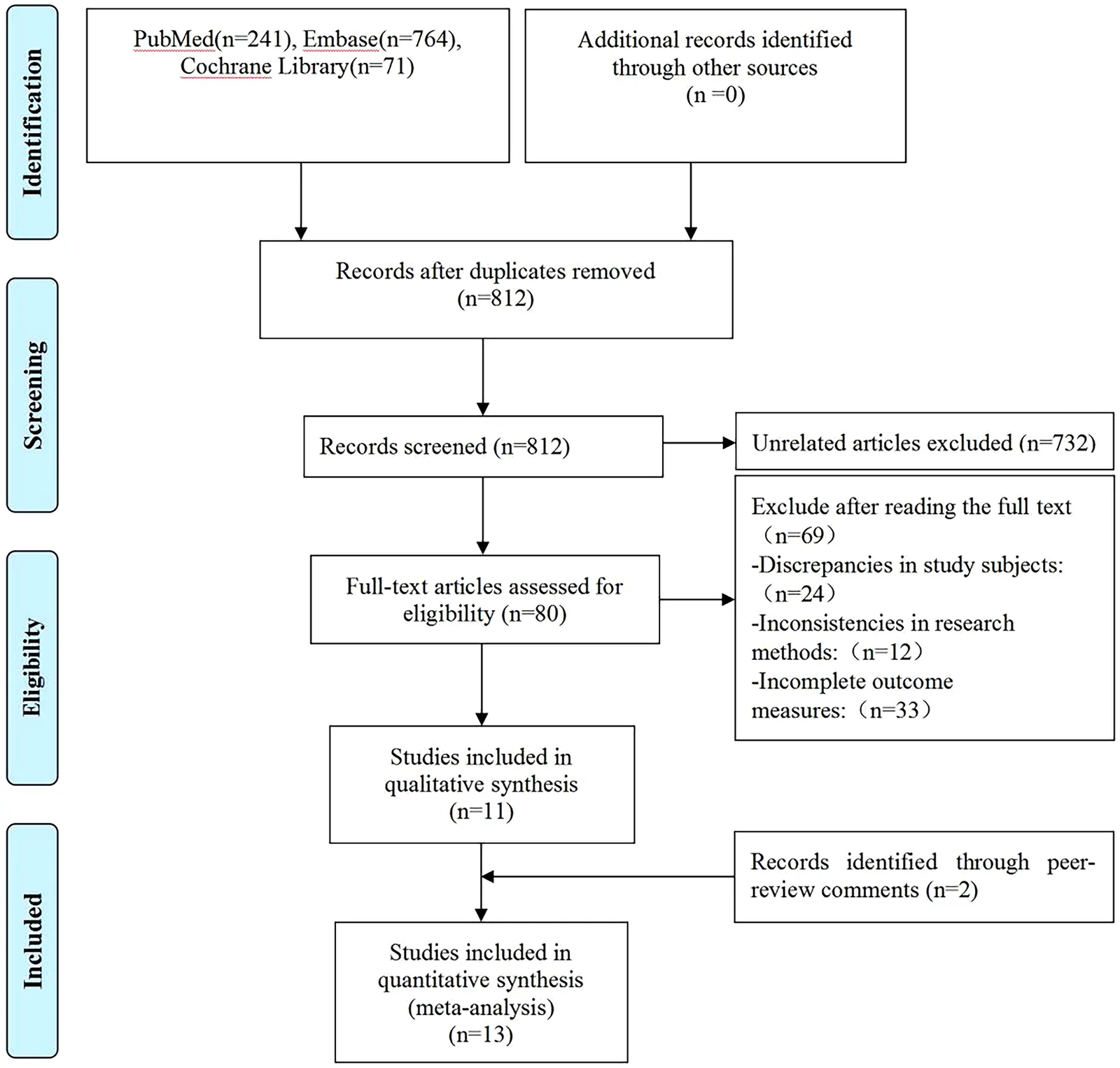
Flowchart of literature screening.

The computerized systematic search of the PubMed, Embase, and Cochrane Library databases yielded 241, 764, and 71 records, respectively, for a total of 1,076. After duplicate removal using reference management software, 812 records remained. Subsequently, preliminary screening based on titles and abstracts excluded 732 records that clearly did not meet the inclusion criteria, leaving 80 records for full-text assessment. After further screening by reviewing full texts, 69 records were excluded for the following reasons: 24 had ineligible study populations, 12 had inconsistent study designs or interventions, and 33 had incomplete outcome data or data unsuitable for pooled analysis. Records identified through peer review process (*n* = 2). Ultimately, 13 studies (5, 10–21) met the inclusion criteria and were included in this meta-analysis. The literature screening process is detailed in Figure 1.

### 3.2 Basic characteristics of included studies

This meta-analysis included 13 studies published between 2015 and 2025, comprising 2 randomized controlled trials (RCTs), 1 cohort study, and 10 non-randomized controlled trials (non-RCTs). The studies were conducted primarily in the United States (6 studies), with the remaining studies from China (2), Germany (2), France (1), Spain (1), and Turkey (1), reflecting geographical diversity. All study populations consisted of patients with acute pulmonary embolism, with disease severity uniformly classified as intermediate-risk PE. The definition of intermediate-risk pulmonary embolism varied slightly across included studies. All studies required hemodynamic stability (absence of persistent hypotension or shock) plus evidence of right ventricular dysfunction (by echocardiography or computed tomography) and/or elevated cardiac biomarkers (troponin, BNP, or NT-proBNP). Among the 13 included studies, 9 studies provided information on the 2019 European Society of Cardiology (ESC) risk substratification (intermediate-high vs. intermediate-low risk). The distribution of patients by ESC risk class is presented in Table 3. The remaining 4 studies did not report ESC risk subcategories, although their inclusion criteria were consistent with the broader intermediate-risk definition.

**Table 3:** Basic characteristics of included studies.

Study	Year	Study Type	Country	Concomitant Anticoagulation (CA)	Intervention (Thrombolytic Agent, Dose, Route)	Sample Size	2019 ESC Risk Class	Outcomes	Follow-up
Victor F. Tapson (10)	2018	RCT	USA	All patients received therapeutic anticoagulation; unfractionated heparin reduced to 300–500 U/h during thrombolytic infusion, increased to full therapeutic dosing after procedure. Low-molecular-weight heparin patients received therapeutic doses.	[CA + UACDLDT: tPA 4 mg per lung (2 mg/h per catheter over 2 h or 1 mg/h over 4 h), *n* = 55] vs. [tPA 6 mg per lung over 6 h (1 mg/h per catheter), *n* = 28] vs. [tPA 12 mg per lung over 6 h (2 mg/h per catheter); unilateral PE: half dose, *n* = 18]	101	Intermediate-high (*n* = 28), Intermediate-low (*n* = 73)	1-month mortality, 1-year mortality, major bleeding, recurrent PE	1 year
Ling-Yun Zhang (5)	2018	RCT	China	Activated partial thromboplastin time checked after recombinant tissue plasminogen activator infusion; if activated partial thromboplastin time <80 s, enoxaparin 1 mg/kg subcutaneously every 12 h; warfarin started on day 1 after enoxaparin injection; enoxaparin stopped after international normalized ratio stabilized at 2.0–3.0 for ≥2 days; warfarin continued for ≥3 months (target international normalized ratio 2.5).	[CA + LDST: Alteplase (rt-PA) 30 mg IV over 2 h, *n* = 33] vs. [CA alone (anticoagulation only), *n* = 33]	66	Intermediate-high (*n* = 23), Intermediate-low (*n* = 43)	Major bleeding, minor bleeding, recurrent PE	3 months
Chuan Jiang (11)	2021	Cohort study	USA	Standard anticoagulation, primarily unfractionated heparin or low-molecular-weight heparin. For ultrasound-assisted catheter-directed thrombolysis patients, unfractionated heparin used during procedure (patients initially on low-molecular-weight heparin converted to unfractionated heparin before ultrasound-assisted catheter-directed thrombolysis).	[CA alone (anticoagulation only), *n* = 158] vs. [CA + LDST: Alteplase 50 mg (10 mg bolus + 40 mg over 2 h), *n* = 22] vs. [CA + FDST: Alteplase 100 mg (10 mg bolus + 90 mg over 2 h), *n* = 30] vs. [CA + UACDLDT: EKOS, Alteplase median 24 mg, median 12 h, *n* = 47]	257	NA	1-month mortality, 1-year mortality, major bleeding, minor bleeding	1 year
Cyrus Moini (12)	2025	Non-RCT	France	Anticoagulation un-protocolized, using low-molecular-weight heparin, unfractionated heparin, or non-vitamin K antagonist oral anticoagulants. In thrombolysis arm, anticoagulation started immediately after tissue plasminogen activator infusion.	[CA + LDST: tPA 50 mg continuous IV over 2 h (no bolus), *n* = 30] vs. [CA alone (anticoagulation only), *n* = 30]	60	Intermediate-high (*n* = 57), Intermediate-low (*n* = 3)	1-month mortality, major bleeding, minor bleeding	1 month
Xiaogang Jing (13)	2018	Non-RCT	China	Low-molecular-weight heparin subcutaneously every 12 h; overlapping oral warfarin started on days 1–3 after low-molecular-weight heparin injection. Low-molecular-weight heparin stopped 4–5 days after warfarin added, once international normalized ratio stabilized at 2.0–3.0. Warfarin continuously used for at least 3–6 months. For thrombolysis groups, anticoagulation followed thrombolytic therapy.	[CA alone (anticoagulation only), *n* = 16 vs. CA + LDST: Urokinase 500,000 IU/day in 100 mL NS, IV drip over 5 h daily for 5–7 days, *n* = 36] vs. [CA + FDST: Urokinase 4,400 IU/kg loading + 4,400 IU/kg/h for 12–24 h, *n* = 14]	66	NA	1-year mortality, major bleeding, minor bleeding, recurrent PE	1 year
Sara Alcántara Carmona (14)	2018	Non-RCT	Spain	Systemic unfractionated heparin anticoagulation started concomitantly and titrated to activated partial thromboplastin time 1.5–2 × control; systemic anticoagulation continued after completion of thrombolysis (continued on the ward after intensive care unit).	CA + UACDLDT: Urokinase 200,000 IU bolus + 100,000 IU/h local infusion (max 48–72 h), *n* = 87	87	Intermediate-high (*n* = 67), Intermediate-low (*n* = 20)	Major bleeding, minor bleeding	1 year
Emily Zientek (15)	2023	Non-RCT	USA	Continuous heparin infusion drip. In thrombolysis group, low-dose tissue plasminogen activator given without concomitant heparin administration during infusion; heparin administered afterward.	[CA + LDST: Alteplase (tPA) 25 or 50 mg IV over 4 h (no concomitant heparin), then heparin, *n* = 6] vs. [CA alone (anticoagulation only), *n* = 6]	12	Intermediate-high (*n* = 12), Intermediate-low (*n* = 0)	Major bleeding, minor bleeding	1 month
Daniel P. Rothschild (16)	2019	Non-RCT	USA	After low-dose systemic thrombolysis, systemic heparin for ≥24 h (heparin dosing/targets not reported).	CA + LDST: Alteplase 50 mg IV over 2 h, *n* = 45	45	Intermediate-high (*n* = 45)	1-month mortality, major bleeding	1 year
Emine Serap Yilmaz (17)	2021	Non-RCT	Turkey	Enoxaparin (1 mg/kg) subcutaneously every 12 h (in recombinant tissue plasminogen activator group, started after activated partial thromboplastin time recovered to <2× control value). Warfarin started simultaneously with enoxaparin in recombinant tissue plasminogen activator group, and 1–2 days later in low-molecular-weight heparin group. Enoxaparin discontinued after ≥5 days when international normalized ratio ≥2.0 for 24 h. Oral anticoagulation continued for ≥3 months.	[CA + LDST: Alteplase (rt-PA) 50 mg in 100 mL saline over 2 h, *n* = 38] vs. [CA alone (anticoagulation only), *n* = 38]	76	NA	1-month mortality, recurrent PE	6 months
Brandon Hooks (18)	2020	Non-RCT	USA	Systemic heparin administered after thrombolysis and continued for at least 24 h.	CA + UACDLDT: EKOS, rt-PA 24 mg (1 mg/h per catheter for 12 h bilateral or 24 h unilateral), *n* = 83	83	NA	1-month mortality, major bleeding, minor bleeding	1 month
Sandeep Bagla (19)	2015	Non-RCT	USA	Concomitant unfractionated heparin during ultrasound-assisted thrombolysis (target activated partial thromboplastin time 60 s); continued for an additional 2 h after thrombolysis, followed by oral anticoagulation.	CA + UACDLDT: EKOS; rt-PA 6 mg per catheter over 6 h (total 6 mg unilateral, 12 mg bilateral), *n* = 45	45	Intermediate-high (*n* = 31), Intermediate-low (*n* = 14)	1-month mortality, major bleeding, minor bleeding, recurrent PE	1 month
Abdelrahman Elhakim (20)	2022	Non-RCT	Germany	Full-dose intravenous unfractionated heparin initiated before EKOS therapy (target partial thromboplastin time 60–80 s), continued during thrombolysis and followed by full therapeutic anticoagulation after procedure.	CA + UACDLDT: EKOS; Alteplase (t-PA) 6 mg over 6 h (unilateral) or 12 mg over 6 h (bilateral), *n* = 41	41	Intermediate-high (*n* = 41), Intermediate-low (*n* = 0)	1-month mortality, major bleeding, minor bleeding, recurrent PE	3 months
Hani Al-Terki (21)	2023	Non-RCT	Germany	Concomitant unfractionated heparin administered during ultrasound-assisted thrombolysis with dosing adjusted to maintain target activated partial thromboplastin time of 60 s; followed by unfractionated heparin for an additional 2 h after thrombolysis, then transitioned to oral anticoagulation.	CA + UACDLDT: EKOS; rt-PA 6 mg per catheter over 6 h (6 mg unilateral, 12 mg bilateral), *n* = 79	79	Intermediate-high (*n* = 79), Intermediate-low (*n* = 0)	1-month mortality, major bleeding, minor bleeding, recurrent PE	1 month

UACDLDT, Ultrasound-assisted catheter-directed low-dose; LDST, Low-dose systemic thrombolysis; FDST, Full-dose systemic thrombolysis. RCT, Randomized Controlled Trial. ESC, European Society of Cardiology. NA, Not available.

### 3.3 Quality assessment of included studies

This meta-analysis included two randomized controlled trials (5, 10). Regarding bias arising from the randomization process, both studies were rated as low risk. For bias due to deviations from intended interventions, both raised concerns, mainly about potential deviations during intervention implementation. The remaining domains—bias due to missing outcome data, bias in measurement of the outcome, and bias in selection of the reported result—were all assessed as low risk. Based on the evaluation across all bias domains, the overall risk of bias for both RCTs was low. See Table 4.

**Table 4:** Quality assessment of randomized controlled trials.

Author	Year	R1	R2	R3	R4	R5	Overall risk of bias
Victor F. Tapson (10)	2018	Low risk	Some concerns	Low risk	Low risk	Low risk	Low risk
Ling-Yun Zhang (5)	2018	Low risk	Some concerns	Low risk	Low risk	Low risk	Low risk

R1: Bias arising from the randomization process; R2: Bias due to deviations from intended interventions; R3: Bias due to missing outcome data; R4: Bias in measurement of the outcome; R5: Bias in selection of the reported result.

The cohort study published by Chuan Jiang et al. (11) scored points for all items, including selection of subjects, definition of exposure, absence of the outcome at baseline, comparability, assessment of outcome, and adequacy of follow-up, achieving a total score of 8 points. This indicates high overall methodological quality and a low risk of bias. See Table 5.

**Table 5:** Quality assessment of cohort studies.

Author	Year	REC	SNEC	AE	DOIS	Comparability		Outcome			Total score
						CMF	COCF	AO	FULO	AFC	
Chuan Jiang (11)	2021	*	*	*	*	*	*	*	*	-	8

REC, Representativeness of the exposed cohort; SNEC, Selection of the non-exposed cohort; AE, Ascertainment of exposure; DOIS, Demonstration that outcome of interest was not present at start of study; CMF, Comparability of major factors; COCF, Comparability of other confounding factors; AO, Assessment of outcome; FULO, Was follow-up long enough for outcomes to occur; AFC, Adequacy of follow-up of cohorts.

Ten non-randomized studies (12–21) were assessed using the ROBINS-I tool. For bias due to confounding, participant selection, and intervention classification, all studies were judged to be at low risk. Regarding bias due to deviations from intended interventions, some studies were rated as moderate risk. Bias in the measurement of the outcome was also mild in some studies. For bias due to missing data, most studies were low risk, with only a few rated as moderate. Bias in the selection of the reported result was low risk in all studies. See Table 6.

**Table 6:** Quality assessment of non-randomized controlled trials.

Author	Year	BC	SP	COI	DFII	MD	MO	SRR	Overall Risk of Bias
Cyrus Moini (12)	2025	Low	Low	Low	Low	Moderate	Moderate	Low	Moderate Risk
Xiaogang Jing (13)	2018	Low	Low	Low	Moderate	Low	Moderate	Low	Moderate Risk
Sara Alcántara Carmona (14)	2018	Low	Low	Low	Moderate	Low	Moderate	Low	Moderate Risk
Emily Zientek (15)	2023	Low	Low	Low	Moderate	Low	Low	Low	Low
Daniel P. Rothschild (16)	2019	Low	Low	Low	Low	Low	Low	Low	Low
Emine Serap Yilmaz (17)	2021	Low	Low	Low	Moderate	Low	Moderate	Low	Moderate Risk
Brandon Hooks (18)	2020	Low	Low	Low	Moderate	Moderate	Low	Low	Moderate Risk
Sandeep Bagla (19)	2015	Low	Low	Low	Low	Low	Low	Low	Low
Abdelrahman Elhakim (20)	2022	Low	Low	Low	Low	Low	Low	Low	Low
Hani Al-Terki (21)	2023	Low	Moderate	Low	Low	Low	Low	Low	Moderate Risk

BC, Bias due to confounding; SP, Selection of participants; COI, Classification of interventions; DFII, Deviations from intended interventions; MD, Missing data; MO, Measurement of outcomes; SRR, Selection of the reported result.

The comprehensive assessment showed that four studies had a low risk of bias, while the remaining six had a moderate risk. No study was found to have a high overall risk of bias.

### 3.4 Efficacy and safety of low-dose thrombolytic therapy in intermediate-risk PE

#### 3.4.1 Patient mortality with low-dose thrombolytic therapy in intermediate-risk PE

Nine studies (10–12, 16–21)reporting 1-month mortality after low-dose thrombolysis for intermediate-risk PE were included, comprising 531 patients with 15 deaths. Heterogeneity testing showed I^2^ = 0.0%, indicating low heterogeneity; therefore, a fixed-effects model was used for meta-analysis. The pooled result demonstrated that the 1-month mortality rate after low-dose thrombolysis in intermediate-risk pulmonary embolism was 2% (95% CI: 0.00–0.03). Subgroup analysis based on the route of thrombolysis administration revealed that in the CA + UACDLDT subgroup, the pooled estimate for the 1-month mortality rate was 2% (95% CI: 0.01–0.04), while in the CA + LDST subgroup, it was a pooled estimate of 1% (95% CI: 0.00–0.03). In addition, three studies (11, 12, 17) reported 1-month mortality in intermediate-risk PE patients treated with conventional anticoagulation alone (CA), including 226 patients with 20 deaths. Using a fixed-effects model, the pooled 1-month mortality rate after CA alone was 8% (95% CI: 0.05–0.13). Moreover, one study (11)reported outcomes for CA plus full-dose systemic thrombolysis (CA + FDST), including 30 patients with 0 deaths; a fixed-effects model yielded a pooled 1-month mortality rate of 0% (95% CI: 0.00–0.12). See Table 7.

**Table 7:** Pooled mortality rates following low-dose thrombolysis, anticoagulation alone, and full-dose thrombolysis.

Category	Subgroup	No. of Studies	Effect Model	ES	95%CI
1-month mortality after low-dose thrombolysis		9	Fixed-effects model	2%	0.00–0.03
	CA + LDST	4	Fixed-effects model	1%	0.00–0.03
	CA + UACDLDT	6	Fixed-effects model	2%	0.01–0.04
1-month mortality after CA		3	Fixed-effects model	8%	0.05–0.13
1-month mortality after full-dose thrombolysis		1	Fixed-effects model	0%	0.00–0.12
1-year mortality after low-dose thrombolysis		3	Fixed-effects model	2%	0.00–0.05
	CA + LDST	2	Fixed-effects model	7%	0.01–0.16
	CA + UACDLDT	2	Fixed-effects model	1%	0.00–0.03
1-year mortality after CA		2	Fixed-effects model	16%	0.10–0.22
1-year mortality after full-dose thrombolysis		2	Fixed-effects model	1%	0.00–0.07

Three studies (10, 11, 13) reported 1-year mortality after low-dose thrombolysis for intermediate-risk PE, comprising 206 patients with 10 deaths. Heterogeneity testing yielded I^2^ = 0.00%, indicating very low heterogeneity; therefore, a fixed-effects model was used for meta-analysis. The pooled result showed that the 1-year mortality rate after low-dose thrombolysis in intermediate-risk pulmonary embolism was 2% (95% CI: 0.00–0.05). Subgroup analysis based on the route of thrombolysis administration revealed that in the CA + UACDLDT subgroup, the pooled estimate for the 1-year mortality rate was 1% (95% CI: 0.00–0.03), while in the CA + LDST subgroup, it was a pooled estimate of 7% (95% CI: 0.01–0.16). In addition, two studies (11, 13) reported 1-year mortality in intermediate-risk PE patients treated with CA alone, including 174 patients with 30 deaths. Using a fixed-effects model, the pooled 1-year mortality rate after CA alone was 16% (95% CI: 0.10–0.22). Furthermore, two studies (11, 13) reported 1-year mortality in patients treated with CA + FDST, including 44 patients with 1 death; a fixed-effects model yielded a pooled 1-year mortality rate of 1% (95% CI: 0.00–0.07). See Table 7.

#### 3.4.2 Patient bleeding incidence with low-dose thrombolytic therapy in intermediate-risk PE

Twelve studies (5, 10–16, 18–21) reported the incidence of major bleeding after low-dose thrombolysis for intermediate-risk PE, involving 655 patients and 18 major bleeding events. Regarding major bleeding after low-dose thrombolysis, the pooled incidence across 12 studies (5, 10–16, 18–21) using a fixed-effects model was 1% (95% CI: 0.00–0.03). In subgroup analyses by treatment modality, the pooled incidence was estimated at 1% (95% CI: 0.00–0.03) in six studies of CA + LDST and estimated at 2% (95% CI: 0.01–0.04) in seven studies of CA + ultrasound-assisted catheter-directed low-dose thrombolysis (UACDLDT). When stratified by thrombolytic dose, the pooled observed rates were estimated to be 0% (95% CI: 0.00–0.18) for alteplase 6 mg; 0% (95% CI: 0.00–0.06) for alteplase 8 mg; 2% (95% CI: 0.00–0.05) for alteplase 12 mg; 3% (95% CI: 0.01–0.06) for alteplase 24 mg; 0% (95% CI: 0.00–0.46) for alteplase 25 or 50 mg; 0% (95% CI: 0.00–0.11) for alteplase 30 mg; and 3% (95% CI: 0.00–0.08) for alteplase 50 mg. Two regimens of urokinase were reported: one study (13) using 500 000 IU/day for 5–7 days yielded a rate of 0% (95% CI: 0.00–0.10), and one study (14) using an initial 200 000 IU bolus followed by 100 000 IU/hour locally for up to 48–72 h gave a rate of 3% (95% CI: 0.01–0.10). In addition, five studies (5, 11–13, 15) reported major bleeding events in intermediate-risk PE patients treated with CA alone, including 243 patients with 5 major bleeding events. Based on these counts, the overall event rate is approximately 2.1% (5/243). Furthermore, two studies (11, 13) reported major bleeding events in patients treated with CA + FDST, including 44 patients with 1 major bleeding event; the pooled incidence was 2% (95% CI: 0.00–0.09). See Table 8.

**Table 8:** Pooled incidence of Major bleeding following Low-dose thrombolysis: overall and stratified by treatment modality and thrombolytic agent dosage.

Category	Subgroup	No. of Studies	Effect Model	ES	95%CI
Total		12	Fixed-effects model	1%	0.00–0.03
Treatment modality					
	CA + LDST	6	Fixed-effects model	1%	0.00–0.03
	CA + UACDLDT	7	Fixed-effects model	2%	0.01–0.04
Thrombolytic agent dosage					
	Alteplase = 6 mg	1	Fixed-effects model	0%	0.00–0.18
	Alteplase = 8 mg	1	Fixed-effects model	0%	0.00–0.06
	Alteplase = 12 mg	3	Fixed-effects model	2%	0.00–0.05
	Alteplase = 24 mg	4	Fixed-effects model	3%	0.01–0.06
	Alteplase = 25 or 50 mg	1	Fixed-effects model	0%	0.00–0.46
	Alteplase = 30 mg	1	Fixed-effects model	0%	0.00–0.11
	Alteplase = 50 mg	3	Fixed-effects model	3%	0.00–0.08
	Urokinase = 500,000 IU/day for 5–7 days	1	Fixed-effects model	0%	0.00–0.10
	Urokinase = Initial 200,000 IU bolus, then 100,000 IU/hour via local infusion. Max treatment duration: 48–72 h	1	Fixed-effects model	3%	0.01–0.10

Given the variability in the definitions of major bleeding across the included studies, a sensitivity analysis was conducted to assess the impact of excluding studies that lacked explicit ISTH or GUSTO criteria for major bleeding. As detailed in Table 2, the studies by Ling-Yun Zhang (5) and Xiaogang Jing (13) did not specify a clear definition for major bleeding. The sensitivity analysis revealed that excluding these studies, individually or collectively, did not substantially alter the pooled incidence of major bleeding among intermediate-risk PE patients within the CA + LDST subgroup. Specifically, the pooled incidence remained stable at 1% (95% CI: 0.00–0.05) after excluding Jing (13), 1% (95% CI: 0.00–0.04) after excluding Zhang (5), and 2% (95% CI: 0.00–0.07) when both were excluded. Therefore, the findings from Zhang (5) and Jing (13) appear to have a negligible influence on the overall major bleeding rate, demonstrating the robustness of our results.

Nine studies (5, 11–15, 18, 19, 21) reported the incidence of minor bleeding after low-dose thrombolysis for intermediate-risk PE, involving 468 patients and 43 minor bleeding events. Regarding minor bleeding after low-dose thrombolysis, the pooled incidence using a fixed-effects model was 7% (95% CI: 0.05–0.10). In subgroup analyses by treatment modality, the pooled incidence was estimated to be 3% (95% CI: 0.00–0.08) in five studies of CA + low-dose systemic thrombolysis (LDST) and estimated to be 9% (95% CI: 0.06–0.12) in five studies of CA + UACDLDT. When stratified by thrombolytic dose, the pooled observed rates were estimated to be 5% (95% CI: 0.00–0.26) for alteplase 6 mg; 13% (95% CI: 0.06–0.22) for alteplase 12 mg; 5% (95% CI: 0.02–0.09) for alteplase 24 mg; 0% (95% CI: 0.00–0.46) for alteplase 25 or 50 mg; 24% (95% CI: 0.11–0.42) for alteplase 30 mg; and 0% (95% CI: 0.00–0.04) for alteplase 50 mg. For urokinase, one study (13) using 500 000 IU/day for 5–7 days yielded a minor bleeding rate of 3% (95% CI: 0.00–0.15), and one study (14) using an initial 200 000 IU bolus followed by 100 000 IU/hour locally for up to 48–72 h gave a rate of 17% (95% CI: 0.10–0.27). In addition, five studies (5, 11–13, 15) reported minor bleeding events in intermediate-risk PE patients treated with CA alone, including 243 patients with 4 minor bleeding events. Based on these counts, the overall event rate is approximately 1.6% (4/243). Furthermore, two studies (11, 13) reported minor bleeding events in patients treated with CA + FDST, including 44 patients with 6 minor bleeding events; a fixed-effects model yielded a pooled incidence of 13% (95% CI: 0.04–0.26). See Table 9.

**Table 9:** Pooled incidence of minor bleeding following low-dose thrombolysis: overall and stratified by treatment modality and thrombolytic agent dosage.

Category	Subgroup	No. of Studies	Effect Model	ES	95% CI
Total		9	Fixed-effects model	7%	0.05–0.10
Treatment modality					
	CA + LDST	5	Fixed-effects model	3%	0.00–0.08
	CA + UACDLDT	5	Fixed-effects model	9%	0.06–0.12
Thrombolytic agent dosage					
	Alteplase = 6 mg	1	Fixed-effects model	5%	0.00–0.26
	Alteplase = 12 mg	1	Fixed-effects model	13%	0.06–0.22
	Alteplase = 24 mg	3	Fixed-effects model	5%	0.02–0.09
	Alteplase = 25 or 50 mg	1	Fixed-effects model	0%	0.00–0.46
	Alteplase = 30 mg	1	Fixed-effects model	24%	0.11–0.42
	Alteplase = 50 mg	2	Fixed-effects model	0%	0.00–0.04
	Urokinase = 500,000 IU/day for 5–7 days	1	Fixed-effects model	3%	0.00–0.15
	Urokinase = Initial 200,000 IU bolus, then 100,000 IU/hour via local infusion. Max treatment duration: 48–72 h	1	Fixed-effects model	17%	0.10–0.27

#### 3.4.3 Patient recurrence rate with low-dose thrombolytic therapy in intermediate-risk PE

Seven studies (5, 10, 13, 17, 19–21) reported the recurrence rate after low-dose thrombolysis for intermediate-risk PE, involving a total of 387 patients with 8 recurrent events. Heterogeneity testing showed I^2^ = 0.00%, indicating low heterogeneity; therefore, a fixed-effects model was used for meta-analysis. The pooled result demonstrated that the recurrence rate after low-dose thrombolysis in intermediate-risk pulmonary embolism was 1% (95% CI: 0.00–0.02). Subgroup analysis based on the route of thrombolysis administration revealed that in the CA + UACDLDT subgroup, the pooled estimate for the recurrence rate was 0% (95% CI: 0.00–0.01), while in the CA + LDST subgroup, it was a pooled estimate of 4% (95% CI: 0.01–0.09). In addition, three studies (5, 13, 17) reported recurrent PE events among intermediate-risk PE patients treated with CA alone, including 87 patients with 5 recurrences. Using a fixed-effects model, the pooled recurrence rate after CA alone was 5% (95% CI: 0.01–0.11). Moreover, one study (13) reported recurrence in patients treated with CA + FDST, including 14 patients with 3 recurrence events; a fixed-effects model yielded a pooled recurrence rate of 21% (95% CI: 0.05–0.51). See Table 10.

**Table 10:** Pooled recurrence rates in patients with intermediate-risk pulmonary embolism according to treatment strategy.

Category	Subgroup	No. of Studies	Effect Model	ES	95%CI
CA		3	Fixed-effects model	5%	0.01–0.11
Low-dose thrombolysis					
	CA + LDST	3	Fixed-effects model	4%	0.01–0.09
	CA + UACDLDT	4	Fixed-effects model	0%	0.00–0.01
CA + FDST（Xiaogang Jing）		1	Fixed-effects model	21%	0.05–0.51

### 3.5 Comparison of efficacy and safety among low-dose, full-dose, and conventional anticoagulation therapy

Regarding 1-month mortality, compared with CA alone, CA + low-dose systemic thrombolysis (LDST) showed no significant difference (RD = −0.03, 95% CI: −0.14 to 0.08, *P* = 0.592). No statistically significant difference was observed between CA+ LDST and CA+ full-dose systemic thrombolysis (FDST) (RD = 0.19, 95% CI: −0.01 to 0.38, *P* = 0.062). In contrast, CA+ ultrasound-assisted catheter-directed low-dose thrombolysis (UACDLDT) significantly reduced 1-month mortality compared with CA alone (RD = −0.09, 95% CI: −0.13 to −0.04; *P* = 0.043). The comparative effect on 1-month mortality between CA + UACDLDT and CA + FDST could not be reliably estimated because no deaths occurred in either group in the single contributing study. See Table 11.

**Table 11:** Comparison of efficacy and safety between low-dose, full-dose, and conventional anticoagulation therapy.

Outcome	Subgroup	No. of Studies	Effect Model	RD	95%CI	*P*
1-month mortality						
	CA + LDST vs. CA	3	Fixed-effects model	−0.03	−0.14–0.08	0.592
	CA + LDST vs. CA + FDST	1	Fixed-effects model	0.19	−0.01–0.38	0.062
	CA + UACDLDT vs. CA	1	Fixed-effects model	−0.09	−0.13−0.04	0.043
	CA + UACDLDT vs. CA + FDST	1	Not applicable	NE	NE	NE
1-year mortality						
	CA + LDST vs. CA	2	Random-effects model	0.06	−0.10–0.22	0.469
	CA + LDST vs. CA + FDST	2	Random-effects model	0.14	−0.29–0.57	0.516
	CA + UACDLDT vs. CA	1	Fixed-effects model	−0.19	−0.28– −0.11	0.067
	CA + UACDLDT vs. CA + FDST	1	Not applicable	NE	NE	NE
Bleeding incidence						
	CA + LDST vs. CA	5	Random-effects model	0.02	−0.04–0.09	0.502
	CA + LDST vs. CA + FDST	2	Random-effects model	−0.23	−0.44– −0.01	0.037
	CA + UACDLDT vs. CA	1	Fixed-effects model	0.04	−0.18–0.25	0.738
	CA + UACDLDT vs. CA + FDST	1	Fixed-effects model	−0.19	−0.30– −0.07	0.001
Recurrence rate						
	CA + LDST vs. CA	3	Fixed-effects model	−0.02	−0.07–0.03	0.483
	CA + LDST vs. CA + FDST	1	Fixed-effects model	−0.07	−0.16–0.01	0.099

Regarding 1-year mortality, there was no significant difference between CA + LDST and CA alone (RD = 0.06, 95% CI: −0.10 to 0.22, *P* = 0.469). No statistically significant difference was observed between CA + LDST and CA + FDST (RD = 0.14, 95% CI: −0.29 to 0.57, *P* = 0.516). Compared with CA alone, CA + UACDLDT significantly reduced 1-year mortality (RD = −0.19, 95% CI: −0.28 to −0.11; *P* = 0.067). The comparative effect on 1-year mortality between CA + UACDLDT and CA + FDST could not be reliably estimated because no deaths occurred in either group in the single contributing study. See Table 11.

Regarding bleeding incidence, there was no significant difference between CA + LDST and CA alone (RD = 0.02, 95% CI: −0.04 to 0.09, *P* = 0.502). However, CA + LDST was associated with a significantly lower bleeding incidence compared with CA + FDST (RD = −0.23, 95% CI: −0.44 to −0.01, *P* = 0.037). CA + UACDLDT showed no significant difference in bleeding incidence compared with CA alone (RD = 0.04, 95% CI: −0.18 to 0.25, *P* = 0.738), but a significantly lower incidence compared with CA + FDST (RD = −0.19, 95% CI: −0.30 to −0.07, *P* = 0.001). See Table 11.

Regarding recurrence rate, there was no significant difference between CA + LDST and CA alone (RD = −0.02, 95% CI: −0.07 to 0.03, *P* = 0.483). No statistically significant difference was observed between CA + LDST and CA + FDST (RD = −0.07, 95% CI: −0.16 to 0.01, *P* = 0.099). See Table 11.

### 3.6 Publication bias

**Figure 2:**
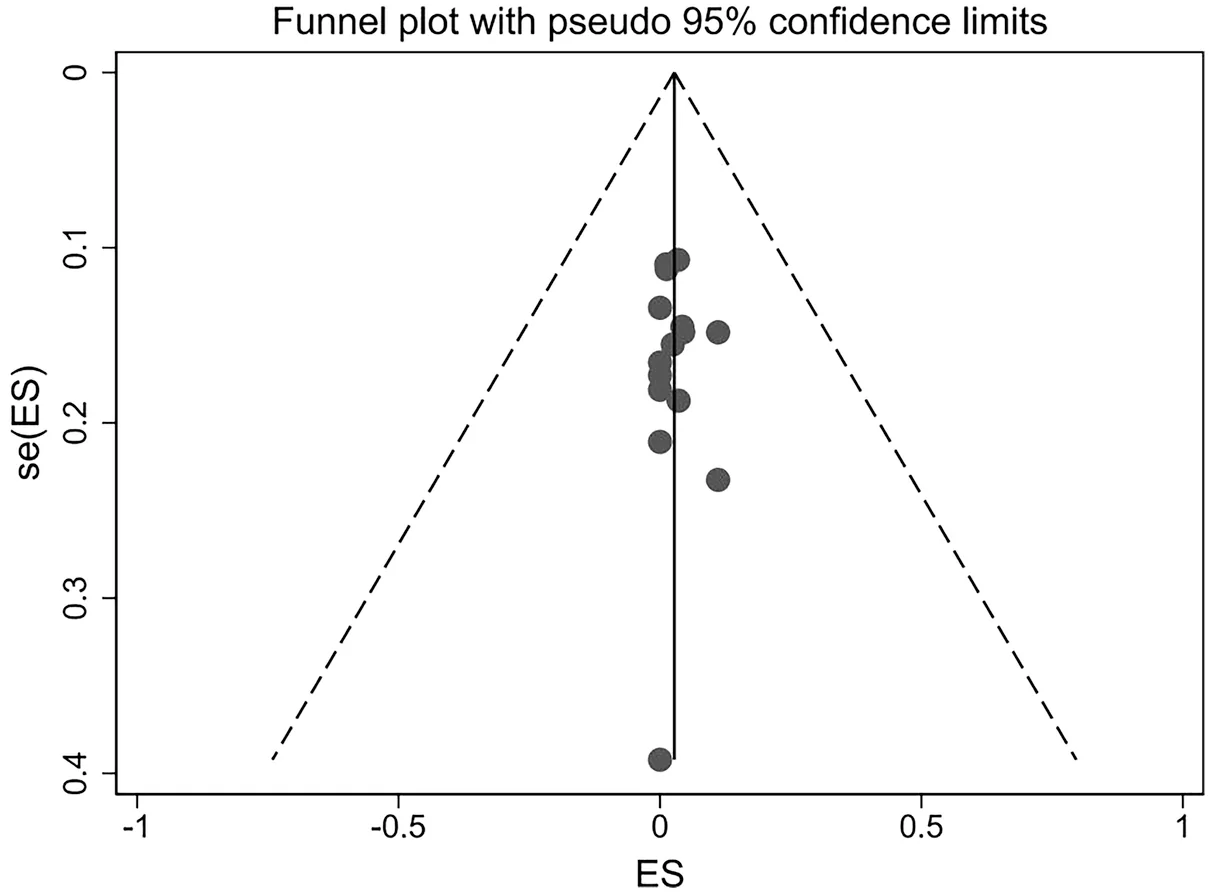
Funnel plot of major bleeding incidence in patients with intermediate-risk PE (*n* = 12).

Twelve studies were included in the analysis of major bleeding; therefore, publication bias for this outcome was assessed using a funnel plot. The funnel plot showed no obvious asymmetry for major bleeding, although publication bias could not be excluded (Figure 2). Formal assessment of publication bias was not performed for 1-month mortality, 1-year mortality, minor bleeding, or recurrence because fewer than 10 studies were available for each outcome.

## 4 Discussion

This meta-analysis indicates that low-dose thrombolysis for intermediate-risk pulmonary embolism demonstrates stable efficacy and acceptable safety. Patients receiving low-dose thrombolysis had low mortality and recurrence rates, without a significant increase in bleeding events. Among low-dose thrombolysis modalities, UACDLDT was associated with a lower 1-year mortality rate, whereas short-term mortality was slightly lower in the LDST subgroup. LDST maintained efficacy with a relatively controlled bleeding risk. These findings suggest that low-dose thrombolysis may achieve a balance between effectiveness and safety in intermediate-risk PE.

Regarding mortality, the 1-month and 1-year mortality rates after low-dose thrombolysis were both 2%. In the UACDLDT subgroup, these rates were 2% and 1%, respectively. This advantage may be attributable to its targeted drug-delivery mechanism: ultrasound-assisted catheter-directed thrombolysis employs acoustic waves to unravel fibrin strands and promote deeper penetration of the thrombolytic agent, thereby maximizing thrombus reduction while minimizing systemic exposure and bleeding risk (22–24). In contrast, conventional LDST may result in incomplete thrombus dissolution in the main pulmonary arteries, potentially explaining higher long-term mortality rates (e.g., 7% at 1 year) (17, 25). However, the finding that UACDLDT significantly reduces 1-month mortality compared with anticoagulation alone (RD = −0.09, 95% CI: −0.13 to −0.04) is based exclusively on a single non-randomized study by Jiang et al. (11). Given the inherent risk of confounding by indication, this finding requires rigorous validation in adequately powered RCTs.

Moreover, these low mortality rates should be interpreted with caution due to potential patient selection bias. The pooled mortality of 2% in our analysis is notably lower than rates reported in broader intermediate-risk PE populations; for instance, Becattini et al. (26) reported an overall 30-day mortality of 7.2% (7.7% in intermediate-high risk, 6.0% in intermediate-low risk). This suggests the included studies may have preferentially enrolled more stable patients, potentially representing a “best-case scenario” rather than the real-world risk profile.

Our analysis did not demonstrate statistically significant differences in mortality or recurrence between LDST plus anticoagulation and anticoagulation alone. This null finding should not be equated with therapeutic equivalence. Baseline short-term mortality in intermediate-risk PE is modest (approximately 3.6%) (27), and detecting between-group differences requires substantial sample sizes that the included small studies lack, making a Type II error possible. Additionally, the 2019 ESC Guidelines subdivide this category into intermediate-high and intermediate-low risk; the admixture of lower- and higher-risk patients would attenuate the true treatment effects observed in the latter subgroup. Furthermore, low-dose regimens offer less fibrinolytic intensity than standard-dose protocols. Collectively, the absence of significant differences is more likely attributable to limitations in sample size, risk stratification, and follow-up design rather than a lack of clinical value.

Regarding recurrence, the rate after low-dose thrombolysis was only 1%. UACDLDT showed no observed recurrence events, whereas LDST had a 4% recurrence rate. This difference may be because UACDLDT better preserves physiological hemodynamics within the pulmonary arteries, lowering the risk of thrombus reformation (22–24, 28, 29), while systemic thrombolysis may leave residual thrombus, contributing to a slightly higher recurrence rate (30–32).

Regarding safety, the pooled incidence of major bleeding following low-dose thrombolysis was 1% (95% CI: 0.00–0.03), which compares favorably with the 6%–10% rates historically reported for full-dose thrombolysis. Both LDST (1%) and UACDLDT (2%) maintained low rates of major bleeding. Minor bleeding was more frequent overall (7%), with higher rates in UACDLDT (9%) than LDST (3%), likely reflecting vascular access requirements, higher baseline bleeding risk in catheter-based patients, and concomitant therapeutic heparin use. Dose-stratified analyses revealed no consistent dose-response relationship between alteplase dose and major bleeding (ranging from 0% to 3% across 6–50 mg). These findings underscore the importance of individualized bleeding risk assessment.

These findings are consistent with the existing literature. While early trials like PEITHO were cautious about systemic thrombolysis due to bleeding risk, the development of low-dose and catheter-directed techniques has been supported by new RCTs (e.g., the SUNSET sPE trial) and observational studies, confirming their dual advantages (33–35). Treatment strategy should be individualized: for patients at high bleeding risk, UACDLDT may be preferred; for patients with ordinary intermediate risk, LDST offers a more straightforward pathway. The ongoing HIPEITHO trial, comparing UACDLDT plus anticoagulation vs. anticoagulation alone in intermediate- to high-risk PE, is expected to provide the highest-level evidence to date and will profoundly shape future clinical decision-making.

Despite these findings, several limitations should be acknowledged: (1) This meta-analysis predominantly included non-randomized studies with limited RCTs, and observational designs cannot entirely avoid confounding factors. (2) Subgroup comparisons, especially regarding UACDLDT, were often based on small-sample studies or single trials, resulting in wide confidence intervals and limited statistical stability. (3) Heterogeneity existed in thrombolytic agents, dosages, administration routes, and concomitant anticoagulation regimens, potentially weakening comparability. (4) Differences in risk stratification criteria, imaging assessment methods, and biomarker choices may mean patients' actual risk levels varied across studies. (5) Data on long-term outcomes, such as RV function recovery and chronic thromboembolic pulmonary hypertension, were limited. (6) Although original studies collected baseline ESC risk class data, they did not report outcomes stratified by intermediate-high vs. intermediate-low risk subgroups, preventing dedicated subgroup analysis for the highest-risk patients. (7) Patient selection bias may limit the generalizability of our findings. The pooled mortality rate in the low-dose thrombolysis group (2%) was considerably lower than the 30-day mortality of 7.2% reported in unselected intermediate-risk PE populations (e.g., Becattini et al. (26)), suggesting that the included studies may have preferentially enrolled hemodynamically more stable patients, representing a “best-case scenario” rather than the full spectrum of real-world intermediate-risk disease. (8)Included studies were predominantly from high-income or upper-middle-income countries, introducing geographic and economic bias; UACDLDT requires advanced infrastructure and specialized personnel that may be unavailable in resource-limited settings, where LDST may be more feasible.

## 5 Conclusion

This meta-analysis suggests that in patients with intermediate-risk pulmonary embolism, low-dose thrombolysis is associated with low overall mortality and recurrence rates, along with an acceptable bleeding risk. Compared with full-dose thrombolysis, low-dose thrombolysis demonstrates certain safety advantages. Efficacy and safety profiles vary across low-dose strategies, with ultrasound-assisted catheter-directed low-dose thrombolysis showing potential benefits for certain outcomes. However, due to the limited number and quality of available studies, the evidence remains constrained, and results should be interpreted with caution. The use of low-dose thrombolysis in intermediate-risk pulmonary embolism still requires individualized risk assessment. Due to the exploratory nature of this meta-analysis and the limitations of the current evidence base, these findings are insufficient to directly guide clinical guideline updates. The primary value of this study lies in generating hypotheses and informing the design of future high-quality randomized controlled trials, which are warranted to validate these findings before any guideline modifications can be considered.

## Data Availability

The original contributions presented in the study are included in the article/Supplementary Material, further inquiries can be directed to the corresponding author/s.

## Glossary

AE: Ascertainment of exposure
AECOPD: Acute exacerbation of chronic obstructive pulmonary disease
AFC: Adequacy of follow-up of cohorts
AO: Assessment of outcome
BC: Bias due to confounding
BNP: B-type natriuretic peptide
CA: Concomitant Anticoagulation
CI: Confidence interval
CMF: Comparability of major factors
COCF: Comparability of other confounding factors
COI: Classification of interventions
DFII: Deviations from intended interventions
DOIS: Demonstration that outcome of interest was not present at start of study
ESC: European Society of Cardiology
FDST: Full-dose systemic thrombolysis
FULO: Was follow-up long enough for outcomes to occur
GUSTO: Global Utilization of Streptokinase and Tissue Plasminogen Activator for Occluded Coronary Arteries
ISTH: International Society on Thrombosis and Hemostasis
IU: International unit
LDST: Low-dose systemic thrombolysis
LMWH: Low-molecular-weight heparin
MD: Missing data
MO: Measurement of outcomes
NOS: Newcastle-Ottawa Scale
NT-proBNP: N-terminal pro-B-type natriuretic peptide
PE: Pulmonary embolism
PRISMA: Preferred Reporting Items for Systematic Reviews and Meta-Analyses
PROSPERO: International Prospective Register of Systematic Reviews
R1: Bias arising from the randomization process
R2: Bias due to deviations from intended interventions
R3: Bias due to missing outcome data
R4: Bias in measurement of the outcome
R5: Bias in selection of the reported result
RCT: Randomized controlled trial
RD: Risk difference
REC: Representativeness of the exposed cohort
RoB 2: Risk of Bias 2 tool
ROBINS-I: Risk Of Bias In Non-randomized Studies of Interventions
RR: Risk ratio
rt-PA: Recombinant tissue plasminogen activator
RV: Right ventricle/Right ventricular
SNEC: Selection of the non-exposed cohort
SP: Selection of participants
SRR: Selection of the reported result
UACDLDT: Ultrasound-assisted catheter-directed low-dose thrombolysis.
